# PimT, an amino acid exporter controls polyene production via secretion of the quorum sensing pimaricin-inducer PI-factor in *Streptomyces natalensis*

**DOI:** 10.1186/1475-2859-8-33

**Published:** 2009-06-08

**Authors:** Cláudia M Vicente, Javier Santos-Aberturas, Susana M Guerra, Tamara D Payero, Juan F Martín, Jesús F Aparicio

**Affiliations:** 1 Instituto de Biotecnología INBIOTEC, 24006 León, Spain; 2 Area de Microbiología, Facultad de Biología, Universidad de León, 24071 León, Spain

## Abstract

**Background:**

Polyenes represent a major class of antifungal agents characterised by the presence of a series of conjugated double bonds in their planar hydroxylated macrolide ring structure. Despite their general interest, very little is known about the factors that modulate their biosynthesis. Among these factors, we have recently discovered a new inducing compound (PI-factor) in the pimaricin producer *Streptomyces natalensis*, which elicits polyene production in a manner characteristic of quorum sensing. Here, we describe the involvement of an amino-acid exporter from *S. natalensis *in modulating the expression of pimaricin biosynthetic genes via secretion of the quorum-sensing pimaricin-inducer PI-factor.

**Results:**

Adjacent to the pimaricin gene cluster lies a member of the RhtB family of amino-acid exporters. Gene deletion and complementation experiments provided evidence for a role for PimT in the export of L-homoserine, L-serine, and L-homoserine lactone. Expression of the gene was shown to be induced by homoserine and by the quorum-sensing pimaricin-inducer PI-factor. Interestingly, the mutant displayed 65% loss of pimaricin production, and also 50% decrease in the production of PI, indicating that PimT is used as PI-factor exporter, and suggesting that the effect in antifungal production might be due to limited secretion of the inducer.

**Conclusion:**

This report describes the involvement of an amino acid exporter (encoded by *pimT *in the vicinity of the pimaricin cluster) in modulating the expression of antibiotic biosynthetic genes via secretion of the quorum-sensing pimaricin-inducer PI-factor. The discovery of the participation of amino acid exporters in a signal transduction cascade for the production of polyene macrolides is unexpected, and represents an important step forward towards understanding the regulatory network for polyene regulation. Additionally, this finding constitutes the first detailed characterization of an amino-acid exporter in an Actinomycete, and to our knowledge, the first evidence for the implication of this type of exporters in quorum sensing.

## Background

Transporters involved in the efflux of low molecular weight substances play important roles in the protection of cells against noxious substances [1], in communication via secretion of regulatory molecules [2], and in the maintenance of an optimal intracellular concentration of metabolites such as sugars [3] and amino acids [4]. Characterization of amino acid efflux systems has been mainly restricted to *Escherichia coli *and *Corynebacterium glutamicum *because they are the main bacteria used for amino acid production at the industrial level (see [5] for a review). Work with these species has permitted the molecular characterization of a series of exporters of amino acids in the last few years. These include exporters like RhtB, LysE, ThrE, and YdeD, each representing the prototype of a different transporter family.

The RhtB transporters can be divided into two subfamilies named RhtB and LysE [6], which are structurally very closely related, and resemble each other in molecular mass, and topology of their membrane-spanning helices, showing however slightly different conserved sequence motifs [6]. LysE from *C. glutamicum *was the first exporter whose gene was cloned [7], and exports basic amino acids like L-lysine and L-arginine at comparable rates [8]. In *E. coli*, RhtB confers resistance to L-homoserine, and L-homoserine lactone, whereas RhtC drives the efflux of L-threonine [9]. YfiK and YeaS from *E. coli *also belong to the RhtB family of export proteins, and promote the export of L-cysteine and *O*-acetylserine [10], and L-leucine [11], respectively.

The *C. glutamicum *ThrE exporter does not belong to the RhtB superfamily: it is larger in size, it shows ten transmembrane-spanning helices [12], and it exports both L-threonine and L-serine [13], whereas the *E. coli *exporter YdeD is implicated in the export of O-acetylserine or L-cysteine and belongs to the PecM family of transporters [14].

Bacteria belonging to the genus *Streptomyces *are well-known for their ability to produce a variety of antibiotics and other secondary metabolites [15]. Production of these compounds is regulated in response to an altered nutritional status [16] and to a variety of environmental conditions, and hence occurs in a growth-phase-dependent manner and usually accompanied by morphological differentiation [17]. Pimaricin is a tetraene macrolide antifungal antibiotic produced by *S. natalensis*. As a polyene, its antifungal activity lies in its interaction with membrane sterols, which alters the membrane structure and leads to the leakage of cellular materials [18]. Like other macrocyclic polyketides, pimaricin is synthesized by the action of so-called type I modular polyketide synthases [19]. Its biosynthetic gene cluster has been characterized [20-27], and some of the factors that modulate its biosynthesis have been identified [28].

Secondary metabolism and cell differentiation in actinomycetes are often controlled by diffusible butyrolactones which act as quorum-sensing signals [29]. Recently, we have identified a novel quorum-sensing inducer (PI-factor; 2,3-diamino -2,3-bis (hydroxymethyl) -1,4-butanediol) which elicits pimaricin production at nanomolar concentrations [30]. Here, we describe for the first time, the cloning, sequencing and detailed characterization of an amino-acid exporter from a Streptomycete, and demonstrate its role as a PI-factor transporter in the pimaricin-producing *S. natalensis*.

## Results

### Cloning of *pimT*

**Figure 1:**
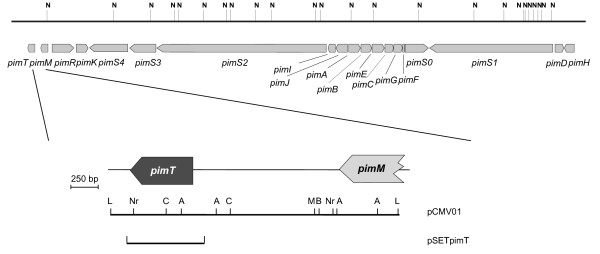
**Pimaricin biosynthetic gene cluster**. The left fringe of the gene cluster is indicated in more detail, and includes *pimT *(in dark grey), and the 3'-end of the transcriptional activator-encoding gene for pimaricin biosynthesis *pimM *[26]. Pointed boxes indicate the direction of transcription. A, *Apa*I; B, *Bam*HI; C,*Acc*I; L, *Apa*LI; M, *Mlu*I; N, *Not*I; Nr, *Nru*I. The inserts used for vector construction are indicated at the bottom.

*pimT *was identified by genomic walking using an *S. natalensis *ATCC 27448 cosmid library [20] and DNA segments from *pimM *(which encodes a pathway-specific regulatory gene for pimaricin production [26]), at the left hand of the pimaricin gene cluster [21]. The gene was sequenced from plasmid pCMV01 (see Methods) and turned out to be separated by 1,366 bp from the 3' end of *pimM*, and in the same orientation (Fig. 1). The initiating ATG codon of *pimT *is preceded by the sequence AGGAGG which could potentially act as a ribosomal binding site. *pimT *is 645 bp long with an overall codon usage pattern in good agreement with that of typical *Streptomyces *genes.

### In silico analysis of the *pimT *gene product

Computer-assisted analysis of the *pimT *gene product (214 amino acids with an estimated M_r _of 22,248) showed a high sequence identity (87.9%) with the whole of protein Orf16 of *Streptomyces hygroscopicus *NRRL 3602, a putative RhtB protein of 227 amino acid residues whose encoding gene was found within the geldanamycin gene cluster [31], and also with SACE 5283 (57.2% identity), a putative LysE protein encoded by the *Saccharopolyspora erythraea *NRRL 2338 genome [32]. Protein database comparisons revealed several additional counterparts, all of them putative RhtB proteins. Interestingly, no homologues were found to be encoded by the *Streptomyces *genomes sequenced up to date, including *S. coelicolor*, *S. avermitilis*, *S. scabies *or *S. griseus*, suggesting that PimT is probably involved in processes related to strain-specific secondary metabolism. PimT analysis revealed that it is highly hydrophobic; it contains six predicted transmembrane helices, and the three conserved sequence signatures of RhtB proteins [6] situated at canonical distances.

### Gene replacement of *pimT*

**Figure 2:**
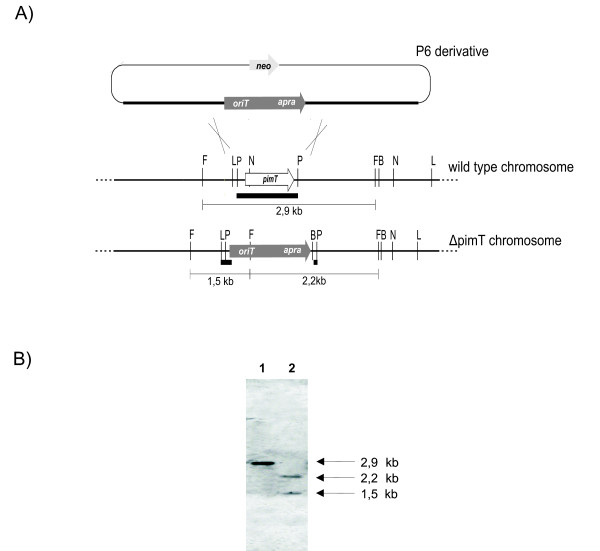
**Gene replacement of *pimT***. A) Predicted restriction enzyme polymorphism caused by gene replacement. The *Afl*III restriction pattern before and after replacement is shown. The probe is indicated by thick lines. B, *Bam*HI; F, *Afl*III; L, *Apa*LI; N, *Nru*I; P, PvuI. B) Southern hybridization of the *Afl*III digested chromosomal DNA of the wild type (lane 1), and Δ*pimT *(lane 2) strains.

In order to determine the function of *pimT*, we inactivated it by using the REDIRECT gene replacement technology as indicated in Methods. Double-crossover mutants were screened by apramycin resistance and kanamycin sensitivity (Fig. 2). These (about 1%) were verified by both PCR and Southern blot analysis. Fig 2 shows the Southern blot of a randomly chosen exconjugant DNA. Chromosomal DNAs isolated from *S. natalensis *ATCC 27448 and mutant Δ*pimT *and digested with *Afl*III were probed with a 809 bp *Pvu*I fragment covering the whole of *pimT *(Fig. 2A). A hybridizing band of 2.9 kb was found for the wild type as expected (Fig. 2B), whereas in the mutant, two hybridizing bands of 2.2 kb and 1.5 kb were observed (Fig. 2B), indicating that a double crossover event had occurred. The observed hybridizing bands corresponded exactly to those expected according to the integration process depicted in Fig. 2.

The new strain *S. natalensis *Δ*pimT *had growth and morphological characteristics identical to those of *S. natalensis *wild type when grown on solid or liquid media, suggesting that PimT has no role in bacterial growth or differentiation. The spore counts of both strains were similar after growth for 9 days at 30°C on TBO plates. The spores of both strains were serially diluted and plated on minimal medium. Both strains grew well in the minimal medium, showing an identical growth curve, which indicates that genes involved in amino acid biosynthesis were not affected.

### Δ*pimT *mutant shows increased sensitivity to L-homoserine, L-serine, and L-homoserine lactone

Some natural amino acids are known to inhibit *E. coli *cell growth when added into minimal media at high concentrations [9,33], and the same phenomenon has been observed in several *Streptomyces *strains (unpublished results). Therefore, the involvement of PimT in the export of amino acids was tested by comparing the resistance of the wild-type strain and the null-mutant for the *pimT *gene to various natural amino acids (Table 1). The ΔpimT mutant was found to have increased sensitivity to L-homoserine (3-fold), L-serine (2-fold) and L-homoserine lactone (1.6-fold) (Table 1). These data suggest that protein PimT assists the export of L-homoserine, L-serine, and L-homoserine lactone out of the cell. No differences in sensitivity were observed when D-homoserine was tested, thus indicating that PimT exhibits stereospecificity for L-homoserine. Also, no differences were observed when other amino acids were tested (Table 1).

**Table 1:** Resistance of *S. natalensis *strains to some amino acids.

Amino acid	Minimal inhibitory concentration (μg/ml)		
	*wt*	Δ*pimT*	Δ*pimT*(pSETpimT)
	
L-Aspartic acid	> 5000	> 5000	
L-Methionine	> 5000	> 5000	
L-Threonine	> 5000	> 5000	
L-Lysine	> 5000	> 5000	
DL-Hydroxynorvaline	180	180	
L-Homoserine	60	20	75
D-Homoserine	> 200	> 200	
L-Homoserine lactone	110	70	81
L-Serine	7400	3700	9000
γ-Butyrolactone	> 5000	> 5000	

Direct measurements of intracellular or extracellular concentrations of PimT amino acid substrates in the wild type and the mutant yielded no detectable differences between the two strains, probably because *S. natalensis *is not an overproducer.

### Transcriptional initiation site and expression of *pimT*

**Figure 3:**
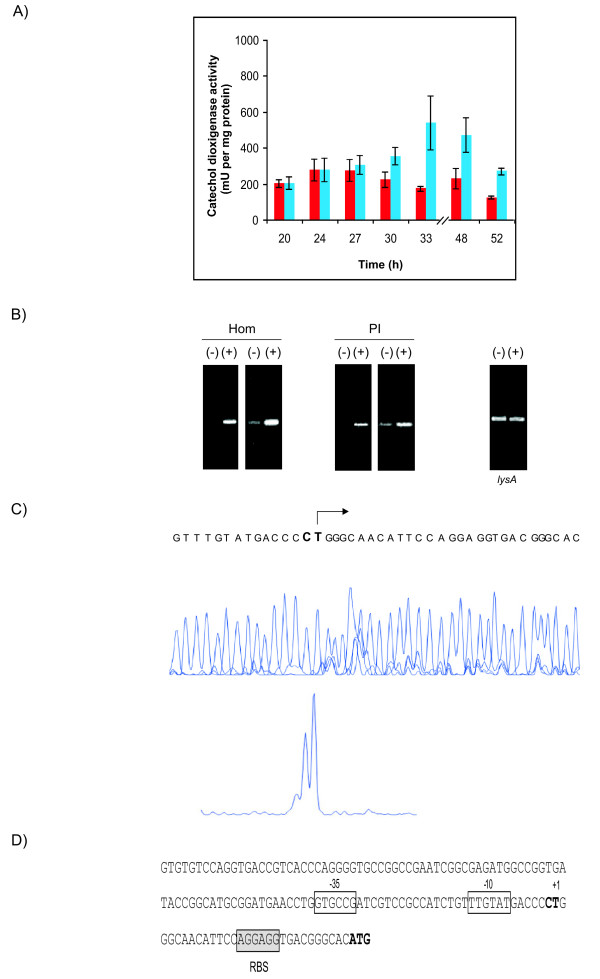
**Promoter activity, gene expression and primer extension analyses**. A) Time course of promoter activity of *pimT *using the *xylE *gene (encoding catechol dioxygenase) as reporter. The enzyme activity of crude extracts was measured at the indicated culture times. Cells were grown in the presence (blue) or absence (red) of added homoserine (2 mg/ml). B) Gene expression analysis of *pimT *by RT-PCR. Analysis was carried out on *S. natalensis *wild type strain in the presence (+) or absence (-) of added homoserine or PI-factor as indicated in the Methods section after 28 (left panels) and 30 (right panels) PCR amplification cycles. The identity of each amplified product was corroborated by direct sequencing. The absence of contaminating DNA in the RNA samples was assessed by PCR. Transcription of the *lysA *gene (encoding diaminopimelate decarboxylase) located outside the *pim *cluster was also assessed as an internal control. The result of the analysis in the absence (-) or presence (+) of homoserine after 30 PCR cycles is shown. Identical result was observed in the presence of PI-factor. C) Mapping of the transcriptional start site of *pimT *by primer extension analysis. The full nucleotide sequence is shown in the upper fluorogram. The nucleotides corresponding to the end of the primer extension are highlighted. The arrow shows the direction of transcription. In the lower panel (D), the start of the transcript is indicated in bold. The -10 and -35 hexanucleotides are boxed, and the first codon is shown in boldface letters. Nucleotides showing homology with the 16S RNA that could form a ribosomal binding site are framed with a box labelled RBS.

To define the transcript initiation site of *pimT*, 143 bp DNA fragment upstream from the ATG start codon was amplified and cloned into the promoter-probe vector pIJ4083 to yield pPpimT (see Methods). This plasmid was used to transform *S. lividans*, and catechol 2,3-dioxygenase activity was measured after growth for several time periods. Expression in YEME medium reached a maximum at 27 h (290 mU/mg protein) and decreased thereafter (Fig. 3A). The same *S. lividans *strain transformed with pIJ4083 (control) yielded no catechol dioxygenase activity. Also, no promoter activity could be detected when we used the successive 152 bp DNA fragment immediately upstream (not shown). The origin of transcription of the *pimT *promoter was determined by several primer extension experiments using a carboxyfluorescein labeled primer (see Methods). These revealed a single transcription start point at 29–30 nucleotides upstream of the ATG translation start site (Fig. 3C). Analysis of the region upstream of the transcription starting site revealed the presence of a -10 box TTGTAT, located at 6 nucleotides from the start site, and a -35 box GTGCCG separated by 16 nucleotides (Fig 3D).

### *pimT *expression is induced by homoserine

It is known that expression of some genes encoding amino acid transporters is induced by their corresponding substrates [8,11]. In order to ascertain whether *pimT *expression was induced by its substrates, we added different concentrations of either L-homoserine, L-serine, or L-homoserine lactone to pPpimT-containing *S. lividans *cultures, and XylE activity of crude extracts was monitored at different time points. These data clearly showed that *pimT *expression was increased substantially by the addition of homoserine (2mg/ml) to the growth medium (Fig. 3A), whereas no differences were observed when we added serine or homoserine lactone (not shown). The increase in expression ranged between 3.1-fold at 33 h of growth to 2.1-fold at 48 h. Also, the expression profile changed when compared with the control culture, reaching its maximum after a longer incubation period (33 h) (Fig. 3A).

To investigate this further, *pimT *expression in the presence and absence of added homoserine was examined in cultures of the *S. natalensis *wild-type strain. Total RNA was prepared from *S. natalensis *wild type after growth for 48 h in YEME medium, in the presence or absence of amino acid (2 mg/ml), and used as template for gene expression analysis by reverse transcriptase-polymerase chain reaction (RT-PCR) as described in the Methods section. Primers for RT-PCR were designed to produce cDNAs of approximately 500 bp. A primer pair designed to amplify a cDNA of the *lysA *gene (encoding diaminopimelate decarboxylase) was used as an internal control. Transcripts were analyzed after 28 and 30 PCR cycles, and analyses were carried out three times for each primer pair. There was a clear increase in the expression level of *pimT *in the presence of added homoserine after both 28 and 30 PCR cycles (Fig. 3B), again consistent with the induction of *pimT *expression by L-homoserine.

### Deletion of *pimT *reduces pimaricin production

**Figure 4:**
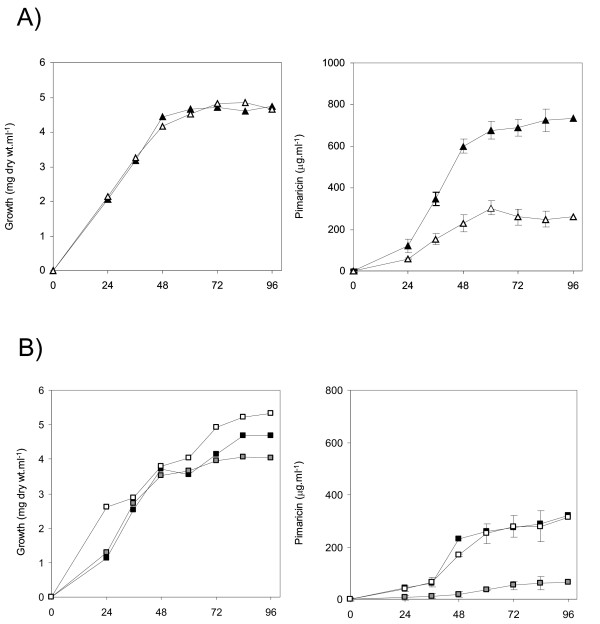
**Replacement of PimT reduces pimaricin production and gene complementation restores antifungal biosynthesis**. Production of pimaricin in YEME medium (right panels). A) solid triangles indicate production by the wild type strain, open triangles the production by the Δ*pimT *strain. B) black squares indicate the production by the wild type strain harboring pSET152neo (control), white squares the production by the Δ*pimT *strain complemented with *pimT*, and grey squares the production by the Δ*pimT *strain harboring pSET152neo. Data are the average of three triplicate flasks. Vertical bars indicate the standard deviation values. Growth curves are shown at the left panels.

The fermentation broth produced by the mutant strain generated by gene replacement, *S. natalensis *ΔpimT, was extracted with butanol and analyzed for the presence of pimaricin. The high performance liquid chromatography (HPLC) analysis indicated that pimaricin production in the mutant strain Δ*pimT*, was only about 35% of the pimaricin accumulated by the wild type strain at 96 h (0.73 g/l) (Fig. 4A). Given that both strains showed identical growth curves, this result prompted further investigation of the possible reasons for the markedly lower pimaricin production.

### Δ*pimT *mutants sustain low production of the quorum-sensing pimaricin-inducer PI-factor

**Figure 5:**
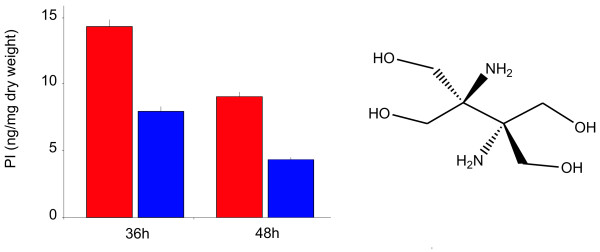
**PI factor production is reduced in the mutant**. Detection was carried out after 36 h and 48 h of growth at 250 rpm and 30°C in YEME medium. Red bars indicate production by the wild type strain, blue bars the production by the Δ*pimT *strain. Pure PI factor was used as standard. Data are the average of three flasks. Error bars indicate the standard deviation values. The structure of the PI factor is indicated.

Given that the majority of members of the RhtB and LysE families export small positively-charged molecules, and that it has been suggested that their physiological role might be to avoid the build-up of substrate compounds to toxic levels in the cytoplasm or to mediate the secretion of signalling molecules [5,6,34], we decided to investigate the involvement of PimT in the export of the quorum-sensing pimaricin inducer PI-factor [30]. PI factor (2,3-diamino-2,3-bis(hydroxymethyl)-1,4-butanediol) (Fig. 5) is a positively charged molecule which might well be an additional substrate for PimT. To analyze the putative involvement of PimT in the efflux of PI factor, *S. natalensis *Δ*pimT *was grown in YEME medium, and extracellular PI factor production was monitored. Only about 50% of the PI factor produced by the wild type could be detected in *S. natalensis *Δ*pimT *cultures after 48 h of growth (Fig. 5). This result suggests that PimT plays a role in PI factor export, and that the levels of PI observed might be due in part to diffusion or to another as yet unidentified carrier. Interestingly, deletion of *pimT *gene increased the intracellular pool of PI. These data (calculated as the mean from three independent experiments) were, respectively, 0.26 and 0.45 nmol/mg dry weight in the wild type strain and the mutant after 48 h of growth. This result indicates that PimT protein influences the accumulation of PI-factor in the medium rather than the synthesis of this inducer in the cell, and strongly suggests that the PI signal must be sensed at the surface of the cell.

### Addition of exogenous PI-factor restores pimaricin production in Δ*pimT *mutants

In order to ascertain whether the reduction of pimaricin production observed in the mutant was due to the substantial decrease in PI-factor export, purified PI-factor was added exogenously to cultures of Δ*pimT *mutants, and antifungal production was monitored. Cells were grown in YEME medium in the presence and absence of 100 nM PI-factor and pimaricin production was assessed. It was found that polyene production in the supplemented cultures was restored to wild type levels (not shown), thus corroborating the role of PimT as PI-factor exporter, and indicating that the extracellular concentration of PI-factor is crucial for pimaricin production.

### *pimT *expression is also induced by PI-factor

In order to test whether *pimT *expression was induced by PI-factor, we added different concentrations of it to pPpimT-containing *S. lividans *cultures, and XylE activity of crude extracts was monitored at different time points. No increase in expression was observed at all (not shown), which is consistent with the signaling role of PI-factor at the membrane level in *S. natalensis*. However, when we studied *pimT *expression in the presence and absence of added PI-factor in cultures of *S. natalensis *wild-type strain by RT-PCR (see above), results showed a clear difference in the expression level of *pimT *in the presence or absence of the inducer after both 28 and 30 PCR cycles (Fig. 3B), being clearly higher in the presence of PI-factor, thus suggesting that *pimT *expression is also induced by PI-factor.

### Gene complementation restores wild type phenotypes

To confirm that the disruption of *pimT *was directly responsible for the reduction of pimaricin production, we complemented the *pimT*-disrupted mutant with *pimT*. A DNA fragment containing *pimT *plus its promoter region was inserted into the integrative vector pSET152neo, giving rise to pSETpimT (see Methods). The plasmid was transferred from *E. coli *ET12567 [pUZ8002] to *S. natalensis *Δ*pimT *by conjugation. pSET152neo was also introduced into *S. natalensis *wild type as control. Introduction of pSETpimT restored pimaricin biosynthesis to the control levels (Fig. 4B). These results were fully consistent with those obtained upon deletion of the *pimT *gene, and confirm the involvement of PimT in pimaricin biosynthesis.

It is noteworthy that production of pimaricin diminished substantially upon introduction of pSET152neo, reaching about 44% of the production observed in the wild type strain without plasmid (Fig. 4A). Similarly, production of the complemented strain, although identical to that of the control strain, was lower than that of the wild type strain bearing no plasmid. Furthermore, introduction of pSET152neo into the mutant also reduced severely pimaricin production, reaching about 25% of the production observed in the mutant strain without plasmid (Fig. 4). This phenomenon is well known in many *Streptomyces *species, and is thought to be derived from the integration of the plasmid into the ΦC31 *attB *site. It has been reported that integration of vectors with an *attP-int *locus from ΦC31 can cause detrimental effects on antibiotic production in some strains [35], and this could be the case of pimaricin and *S. natalensis*. Moreover, it has been reported that pSET152 vectors can integrate into pseudo-*attB *sites in both *S. coelicolor *and *S. lividans *[36] and this could also be happening in *S. natalensis*, thus affecting pimaricin production.

Interestingly, gene complementation also restored PI-factor production to wild-type levels thus confirming the role of the gene product in PI factor secretion. Similarly, the complemented strain also recovered the resistance levels of the wild type to L-homoserine, L-serine, and L-homoserine lactone (Table 1).

## Discussion

Sequencing of the left-hand side of the pimaricin gene cluster in *S. natalensis *revealed the presence of a gene, *pimT*, whose product was found to display six predicted transmembrane helices and was strikingly similar to proteins of the RhtB family of amino acid exporters. Members of this family of exporters are widespread in eubacteria and archaea [6], and most prokaryotic genomes contain a significant number of genes devoted to them [37], but despite their interest only a few have so far been studied in detail. The members of this family that have been characterized mostly belong to *C. glutamicum *or *E. coli *[9-11,33], most likely because these are the two species used in the industrial production of amino acids [34]. Strikingly, no homologues were found to be encoded by any of the *Streptomyces *spp. genomes that have been sequenced up to date. This fact is unexpected for a gene whose proposed function is to achieve homeostasis of the intracellular amino acid concentration, and suggests that the biological function of this gene may not be restricted only to amino acid transport and might be related to strain-specific secondary metabolite biosynthesis and regulation. The lack of studies in *Streptomyces *spp. prompted us to study the involvement of this gene product in the export of amino acids and/or secondary metabolism in *S. natalensis*.

Comparison of the resistance of the wild-type, the ΔpimT strain, and the complemented strain to various amino acids provided evidence for a role of *pimT *in the excretion of L-homoserine, L-serine, and L-homoserine lactone. In Gram negative bacteria N-acyl-homoserine lactones serve as signals that mediate cell to cell communication in concert with cell density [38]. Such quorum sensing systems regulate a variety of global cellular response processes such as, e.g., bioluminescence in *Vibrio*, or virulence in *Pseudomonas aeruginosa *and other pathogens, among others (see [39] for a review). Interestingly, homologues of PimT such as RhtB, have been described to be involved in the efflux of homoserine lactone in *E. coli *[9] and based on this their implication in quorum sensing has been suggested [37]. Our present data demonstrate the involvement of an RhtB amino acid exporter in quorum sensing through the secretion of not only homoserine lactone, but also the quorum sensing pimaricin-inducer PI-factor [30]. This factor triggers pimaricin production in *S. natalensis *mutants that had lost their ability to produce pimaricin in a manner characteristic of quorum sensing [30]. A factor, a well-known *S. griseus *autoregulator of the butyrolactone class [40], has the same effect on *S. natalensis *[30], but whereas A-factor recognition has been described to take place intracellularly [40], our results indicate that PI-factor must be recognized at the membrane level. Given that *S. natalensis *is unable to synthesize A-factor [30], it appears that this strain is able to integrate foreign quorum signals. Moreover, the existence of a gene in *S. natalensis *encoding a butyrolactone-receptor protein [41] has recently been reported. This sensing of "foreign" quorum-sensing signals might be a general phenomenon in bacteria, since *E. coli *is able to respond to different quorum-sensing signals without producing them [42].

The substantial decrease of PI factor release upon deletion of the *pimT *gene explains the low pimaricin production observed in the mutant strain. Conversely, the complemented strain shows a recovery in the production of extracellular PI factor and also of pimaricin, thus suggesting that the PI signal must be sensed at the surface of the cell. Taken together, these results constitute strong evidence for the implication of PimT in the efflux of the inducer. Interestingly, the *S. natalensis *Δ*pimT *mutant still excreted some PI factor out of the cell. Such efflux might be due to diffusion, although the participation of other carriers cannot be excluded.

As already indicated (see above) the detrimental effect on pimaricin production observed upon introduction of the pSET152-derived vector used in this study could be explained by the use of the *attP-int *locus from ΦC31 [35,36]. In this regard, we have also observed that introduction of pSET152-derived vectors into the model Streptomycete *S. coelicolor *A3(2) causes a reduction in the production of the polyketide antibiotic actinorhodin (unpublished results). Along with this, the use of vector pTO1, an integrative plasmid which uses the integrative functions of phage ΦC31, has also been described to reduce the production of bialaphos in the producing strain *S. hygroscopicus *ATCC 21705 [43]. Thus, it seems that this phenomenon is not specific to *S. natalensis *and pimaricin. Hence, it is conceivable that the use of vectors with different integrative functions will overcome such problem. Bearing these results in mind, it is likely that the reported increment in pimaricin production upon gene duplication of the PAS regulator *pimM *in the *S. natale*nsis wild type strain by means of the pSET152-derived vector pSETpimM [26] is actually underestimated, and the actual effect of gene duplication could well be substantially higher than that previously reported by us.

The proteins of the RhtB family are among the most widespread membrane proteins. Most prokaryotic genomes contain a significant number of genes (up to eighteen) devoted to them [37]. As examples, *E. coli *has as many as five paralogues [5], whereas *Saccharopolyspora erythraea*, a Gram positive bacterium, has ten [32]. This fact gives an idea of the importance of the physiological role of these proteins for the cell. However, the significance of the excretion of amino acids is still unclear. Most of the RhtB exporters studied are characterized by relatively wide substrate specificity, and this aspect led to the suggestion that amino acids are accidental substrates for these proteins [5,8]. Whether these proteins export amino acids because of the low specificity of these carriers for their true substrates [8] or because their actual substrates are the amino acids they transport [37] is a matter of debate. In any case, it has been suggested that these systems might be involved in quorum sensing [6], and the role of PimT as an exporter of the quorum sensing pimaricin-inducer PI-factor provides clear evidence for this idea.

## Conclusion

Polyenes represent a major class of antifungal agents characterised by the presence of a series of conjugated double bonds in their planar hydroxylated macrolide ring structure. Despite their general interest, very little is known about the factors that modulate their biosynthesis. This report describes the involvement of an amino acid exporter (encoded by *pimT *in the vicinity of the pimaricin cluster) in modulating the expression of antibiotic biosynthetic genes via secretion of the quorum-sensing pimaricin-inducer PI-factor. The discovery of the participation of amino acid exporters in a signal transduction cascade for the production of polyene macrolides is unexpected, and represents an important step forward towards understanding the regulatory network for polyene regulation. Additionally, to our knowledge, this finding provides the first evidence for the implication of this type of exporters in quorum sensing.

## Methods

### Bacterial strains, cloning vectors and cultivation

*S. natalensis *ATCC 27448 was routinely grown in YEME medium [44] without sucrose. Sporulation was achieved in TBO medium [20]. For pimaricin and PI-factor production, the strains were grown in YEME without sucrose at 30°C and 250 rpm. *Escherichia coli *strain XL1-Blue MR (Stratagene) was used as a host for plasmid subcloning in plasmids pBluescript (Stratagene), pUC18 and pUC19. *E. coli *ET12567 [pUZ8002] was used as donor in intergeneric conjugations. *E. coli *BW25113 was used as the host for Red recombination [45] and to propagate plasmid pIJ790 [46]. *S. lividans *66 [44] was used for promoter activity assessment. The promoter-probe vector used was pIJ4083 which contains a promoterless *xylE *gene [44]. pSETneo is a pSET152 [47] derivative constructed as follows: pTC192-km [48] was digested with *Dra*I and *Ecl*136II and religated to yield pMB1neo; this plasmid was subsequently digested with *Eco*RI, end-filled with Klenow, and religated to eliminate the unique *Eco*RI site, and then cut with *Bam*HI to generate a 913 bp fragment containing the *neo *resistance gene which was cloned into the *Bam*HI site of pSET152. Minimal inhibitory concentrations (MICs) of amino acids were determined on *Streptomyces *MM agar plates [44] containing different concentrations of amino acid. The plates were spotted with 200 viable spores and the MIC was determined after 5 days incubation at 30°C.

### Genetic procedures

Standard genetic techniques with *E. coli *and *in vitro *DNA manipulations were as described by Sambrook and Russell [49]. Recombinant DNA techniques in *Streptomyces *species and isolation of *Streptomyces *total DNA were performed as previously described [44]. Southern hybridization was carried out with probes labeled with digoxigenin by using the DIG DNA labeling kit (Roche Biochemicals). Intergeneric conjugation between *E. coli *ET12567 [pUZ8002] and *S. natalensis *was performed as described [50].

### DNA sequencing and analysis

A 2,661 bp *Apa*LI fragment encompassing the entire *pimT *gene, and the 3'-terminal end of the *pimM *gene (Fig. 1) was blunt-ended with Klenow and cloned into a *Sma*I-cut pUC19 vector to yield pCMV01. This plasmid was then used as a source of DNA for sequencing. Sequencing templates were obtained by random subcloning of fragments generated by controlled partial *Hae*III digestions. DNA sequencing was accomplished by the dideoxynucleotide chain-termination method using the Perkin Elmer Amplitaq Gold Big Dye-terminator sequencing system on double-stranded DNA templates with an Applied Biosystems ABI 3130 DNA genetic analyzer (Foster City, California, USA). Each nucleotide was sequenced a minimum of three times on both strands. Alignment of sequence contigs was performed using the DNA Star program Seqman (Madison, Wis.). DNA and protein sequences were analyzed with the NCBI World Wide Web BLAST server.

### Construction of a ΔpimT mutant

Deletion of *pimT *of *S. natalensis *was made by replacing the wild-type gene with a cassette containing an apramycin selective marker using a PCR based system [46]. The plasmid pIJ773 containing the apramycin resistance gene (*aac*(3)IV) and the *oriT *replication origin was used as a template. The mutant was constructed using the oligonucleotides 5'-**tgaccgctgggcaagattccaggaggtgacgggcacatg**ATTCCGGGGATCCGTCGACC-3' and 5'-*ggcgccgcgacgccgcggagatccgtcagaagcgcccta*TGTAGGCTGGAGCTGCTTC-3' as the forward and reverse primers respectively (the sequence identical to the DNA segment upstream from the start codon of *pimT *is in bold and in lower case and the sequence identical to the segment downstream from the stop codon of *pimT *is in lower case italics). These two long PCR primers (59 nt and 58 nt) were designed to produce a deletion of *pimT *just after its start codon leaving only its stop codon behind. The 3' sequence of each primer matches the right or left end of the disruption cassette (the sequence is shown uppercase in both primers). The extended resistance cassette was amplified by PCR and *E. coli *BW25113/pIJ790 bearing cosmid P6 [20] was electro-transformed with this cassette. The isolated mutant cosmid was introduced into non-methylating *E. coli *ET12567 containing the RP4 derivative pUZ8002. The mutant cosmid was then transferred to *S. natalensis *by intergeneric conjugation [50]. Double cross-over exconjugants were screened for their kanamycin sensitivity and apramycin resistance.

### Complementation of *pimT*

In order to complement the Δ*pimT *replacement mutant, a 924 bp DNA fragment containing the entire *pimT *gene including its own promoter was amplified by PCR with primers Ft (5'-**GAATTC**GTGTGTCCAGGTGACCGTC-3') and Rt (5'-CGT**GAATTC**GATGCCGAGCGC-3'). The PCR product was digested with *Eco*RI and ligated into an *Eco*RI-cut pSET152neo (Am^R^, Km^R^, pUC18 replicon, ΦC31 *attP*), to yield pSETpimT. This plasmid was then transferred by conjugation from *E. coli *ET12567 [pUZ8002] to the *S. natalensis *ΔPimT mutant as previously described [50].

### Promoter activity assessment

To assess the activity of the putative *pimT *promoter a 143 bp DNA fragment upstream from the ATG start codon was amplified by PCR using primers (5'-**GGATCC**GTGCCCGTCACCTCC-3') and (5'-**GAATTC**GTGTGTCCAGGTGACCGTC-3'). The PCR product was digested with *Bam*HI and *Eco*RI and cloned in the same sites of pIJ4083 to yield pPpimT, which was subsequently transformed into *S. lividans *66 [44]. For promoter activity studies approximately 1 × 10^6 ^*S. lividans *spores were pre-germinated in 2 × YT liquid medium for 8 h at 30°C. Germinated spores were harvested by centrifugation, resuspended in YEME medium and used to inoculate 100 ml of the same medium. Thiostrepton (5 μg/ml) and kanamycin (50 μg/ml) were added as selective antibiotics. Catechol 2,3-dioxygenase activity of the *xylE *reporter gene was measured as described by Kieser *et al*.[44].

### Primer extension

Total RNA was isolated from *S. lividans *harbouring pPpimT as described [51]. For primer extension experiments, 16 μg RNA was hybridized to 20 pmol 6FAM-labelled primer (5'-CACATGGCCCGGTCGCATTACAC-3') complementary to the 5'-coding region of *xylE*. Reverse transcription was carried out with Superscript III (Invitrogen), and the products loaded into an ABI 3130 DNA genetic analyzer and analysed with the Gene Mapper^® ^(Applied Biosystems) program.

### Isolation of total RNA

*S. natalensis *ATCC 27448 was grown for 48 h in YEME medium (stationary phase of growth), the cultures were then mixed with one volume 40% (v/v) glycerol, and mycelia were harvested by centrifugation and immediately frozen by immersion in liquid nitrogen. Frozen mycelium was then broken by shearing in a mortar, and the frozen lysate was added to buffer RLT (Qiagen) in the presence of 1.5% (v/v) β-mercaptoethanol. RNeasy Mini Spin columns were used for RNA isolation according to manufacturer's instructions. RNA preparations were treated with DNase I RNase-free (Promega) in order to eliminate possible chromosomal DNA contamination.

### Gene expression analysis by RT-PCR

Transcription was studied by using the SuperScript™ One-Step RT-PCR system with Platinum^® ^Taq DNA polymerase (Invitrogen), using 20 ng of total RNA as template. Conditions were as follows: first strand cDNA synthesis, 50°C for 30 min followed by heating at 94°C for 2 min; amplification, 28 or 30 cycles of 98°C for 15 sec, 64 or 69°C (depending of the set of primers used) for 30 sec, and 72°C for 1 min]. Primers were designed to generate PCR products of approximately 500 bp. The primers used for the detection of *pimT *transcripts were: PIMTS, 5'-GTCGTCGGCAACCTCATCGGCTCATAC-3'; and PIMTAS, 5'-GCGCCCAGGCCCCACAGA-3'. The primers used to amplify a cDNA of the *lysA *gene (encoding diaminopimelate decarboxylase) were: LYSAS, 5'-CGCCCGCCCACAGCAGGTCTTC-3'; and LYSAAS, 5'- TGGGGGTGCATGAGGAACTGAT-3'. Negative controls were carried out with each set of primers and Platinum^® ^Taq DNA polymerase in order to confirm the absence of contaminating DNA in the RNA preparations. The identity of each amplified product was corroborated by direct sequencing of the PCR product.

### Assay of pimaricin production

To assay pimaricin in culture broths, 0.5 ml of culture was extracted with 4 ml of butanol, and the organic phase was diluted in water-saturated butanol to bring the absorbance at 319 nm in the range of 0.1 to 0.4 units. Control solutions of pure pimaricin (Sigma) were used as control. To confirm the identity of pimaricin, an UV-visible absorption spectrum (absorption peaks at 319, 304, 291 and 281 nm) was routinely determined in a Hitachi U-2900 spectrophotometer. Quantitative determination of pimaricin was performed as previously described [52].

### Determination of PI factor

Quantitative determination of PI factor was carried out by reverse phase HPLC using a Waters 600 unit coupled to a PDA 996 detector equipped with a Polarity dC18 column (3.9 × 150 mm; particle size, 5 μm) after derivatization with FMOC (fluorenylmethyl chloroformate) [53]. The PI factor elutes at a retention time of 9.3 min using a mobile phase mixture consisting of a gradient (1.5 ml/min) of acetonitrile in 50 mM sodium acetate pH 4.2 (acetonitrile concentration: 25% for 2 min, up to 75% 2–13 min, up to 100% 13–14 min, 100% 14–18 min, down to 0% 18–19 min, 0% 19–23 min). Pure PI factor [30] was used as standard. Intracellular PI-factor was extracted as described elsewhere [52].

### Nucleotide sequence accession number

The sequence reported here has been deposited in the GenBank database under the accession number FM864219.

## Competing interests

The authors declare that they have no competing interests.

## Authors' contributions

CMV created mutant strain, mapped the transcriptional start site and studied *pimT *expression. JSA and SMG carried out liquid culture experiments and assisted in PI-factor purification. TDP performed gene cloning and complementation experiments. JFM participated in the design of the study and assisted in manuscript writing. JFA conceived and supervised the study, reviewed results and drafted the manuscript. All authors have read and approved the final manuscript.
